# Ethnographic methods for process evaluations of complex health behaviour interventions

**DOI:** 10.1186/s13063-016-1340-2

**Published:** 2016-05-04

**Authors:** Sarah Morgan-Trimmer, Fiona Wood

**Affiliations:** Psychology Applied to Health Group, University of Exeter Medical School, St Luke’s Campus, Heavitree Road, Exeter, EX1 2LU UK; Division of Population Medicine, Cardiff University School of Medicine, Neuadd Meirionnydd, Heath Park, Cardiff, CF14 4YS UK

**Keywords:** Complex interventions, Qualitative research, Ethnography, Randomised controlled trials

## Abstract

This article outlines the contribution that ethnography could make to process evaluations for trials of complex health-behaviour interventions. Process evaluations are increasingly used to examine how health-behaviour interventions operate to produce outcomes and often employ qualitative methods to do this. Ethnography shares commonalities with the qualitative methods currently used in health-behaviour evaluations but has a distinctive approach over and above these methods. It is an overlooked methodology in trials of complex health-behaviour interventions that has much to contribute to the understanding of how interventions work. These benefits are discussed here with respect to three strengths of ethnographic methodology: (1) producing valid data, (2) understanding data within social contexts, and (3) building theory productively. The limitations of ethnography within the context of process evaluations are also discussed.

## Background

Qualitative methods are increasingly used in randomised controlled trials (RCTs) of complex health-behaviour interventions at the various stages of complex intervention development and evaluation, including process evaluation [1, 2]. Qualitative methods can inform the understanding of a problem, the development of an intervention, and the understanding of how an intervention is delivered by agencies and received by participants. In process evaluations, qualitative data can contribute insights into how interventions operate and how outcomes are reached, although in practice, qualitative research is not always used to inform the trials they are part of [1]. Interviews and focus groups are commonly used qualitative methods in process evaluations; for example, they are often used to explore the acceptability of an intervention to participants [3]. Ethnography is a methodology which largely, though not exclusively, employs qualitative methods; however, it has a distinctive approach over and above the particular methods it employs, which could be useful in process evaluations to explore the detail of how complex interventions operate. Despite its benefits, the potential contribution of ethnography to process evaluation has not been realised. This article briefly introduces ethnography as a methodology and then discusses three useful features that are relevant to process evaluations: (1) producing valid data, (2) understanding data within social contexts, and (3) building theory productively. The comments made in this article could be applicable to other types of complex interventions besides those targeting health-behaviour change. The focus here on health-behaviour change and public health is because there is an increasing recognition of the social determinants of health in public health research; studies are consequently addressing the social, environmental, and organisational contexts to a greater degree. Ethnography has traditionally examined social contexts and is, therefore, a very relevant methodology for this field.

Ethnography is characterised by long-term participant observation as a central method, where the researcher spends an extended period of time in a social group in order to collect data. The term ‘ethnography’ is often used interchangeably with the term ‘participant observation’, but it is actually a much broader methodology than this, both because of the range of methods it employs and because it encompasses an overall orientation to research, which is detailed below. It comprises a collection of different ways of eliciting and collecting data, including the observation of individuals and groups of individuals, unstructured interviews, documentary analysis, and the use of a researcher’s field notes. It employs these methods within a long-term, holistic, and flexible approach to data collection:

*The ethnographic researcher participates, overtly or covertly, in people’s daily lives for an extended period of time, watching what happens, listening to what is said, asking questions; in fact collecting whatever data are available to throw light on the issues with which he or she is concerned* [4]*.*

Engagement with a particular social or cultural group is also a distinguishing feature of ethnography, as reflected in Curry et al.’s definition:

*Ethnography is a form of field research that seeks to learn the culture of a particular setting or environment. It often relies on participant observation through prolonged field work and may include other qualitative and quantitative methods. The researcher becomes embedded in ongoing relationships with research participants for the purpose of observing and recording talk and behavior. In such cases, the researcher (as opposed to, for instance, surveys or questionnaires) is the primary instrument for data collection and analysis. The researcher seeks to place specific events into a broader, more meaningful context, with a focus on the culture and social interaction of the observed people or groups. Ethnography is particularly valuable in understanding the influence of social and cultural norms on the effectiveness of health interventions* [5]*.*

Through living and working with communities through extended periods of time, often months or years, ethnographers aim to see and describe the world through the eyes of members of that community. They pay particular attention to the everyday life, narratives of events, social interactions, and the cultural meanings and practices of a community. Ethnographies, unlike some observation studies, are of a social group and are often geographically bound. One exception, however, would be a digital ethnography which explores an online social group. The social group could be a class of school children, a choir, a general practice, or scientists working within a laboratory: the key requirement of the social group is that they share a common culture within the environment in which they are being studied (children in a class may have a different home cultures, but while they are in the class, they share in the culture of the class). The term ‘ethnography’ also refers to the product of the research, as well as the method: it is a distinct piece of writing which summarises an ethnographic study of a particular community or people, usually by an anthropologist. For examples of classic ethnographies, see ‘The Forest People’ [6] or ‘The Innocent Anthropologist’ [7], which describe and explain the social worlds of tribes in ‘exotic’ locations for Western audiences. As well as ethnographic monographs, outputs of ethnography can also include images, particularly in the field of visual ethnography [8].

Ethnographic methods developed within the field of social anthropology in the nineteenth and twentieth centuries. Among the most notable studies, under what became to be known as the ‘British School’ of social anthropology, are those by Bronislaw Malinowski and E. E. Evans-Pritchard [9, 10]. These studies involved long periods of intensive fieldwork and participant observation with small tribes of indigenous people and advocated an approach to anthropology which sought to understand the culture from their perspective. Whereas anthropology had traditionally visited discrete communities in remote areas of the globe, the ‘Chicago School’ of sociology and criminology in the mid-twentieth century adapted the commitments of the early social anthropologists, turning attention to social problems within urban settings closer (geographically) to home. For example, in their study of ‘Street Corner Society’ (first published in 1943), Whyte and colleagues produced vivid portraits of city life by recording the social worlds of street gangs [11]. Two decades later, sociologists from the Chicago School undertook observations of groups of health professionals and healthcare organisations. The most notable of these, perhaps, includes a participant observation of hospital life conducted by Roth in 1963, in which Roth himself was admitted as a patient for tuberculosis (TB) [12], and a study of the transition of novice medical students to aspiring doctors, which was conducted by Howard Becker and colleagues in 1961 [13]. More recent commentaries on the use of ethnography in the health field are available [14–17], as well as ethnographies of health-service professionals and organisations [18–20] and settings such as intensive care units [21]. Studies in medical anthropology have also explored health and illness in different cultures more broadly in terms of how well-being, physical health and mental illness are defined, their meaning, and how symptoms are experienced from the perspectives of people and communities themselves [22]. However, ethnography has been relatively underused in trials, health psychology, and social science research on health behaviour change [23].

Ethnography is a rich and detailed methodology and, thus, well suited to the challenges of understanding how complex interventions work; process evaluations of health-behaviour interventions could therefore benefit from adopting ethnographic methods. Process evaluations complement traditional randomised controlled trials (RCTs) by attempting to understand how interventions operate. They illuminate the ‘black box’ of the processes of an intervention, which are not addressed by the classic RCT design that examines the relationship between a limited set of variables (at baseline and outcome and in intervention and control groups). The recent Medical Research Council (MRC) guidance [24] has conceptualised process evaluation in terms of examining three principal elements: the delivery of the intervention, the mechanisms of impact that occur in participants (how changes in individuals’ health behaviours occur), and contextual factors which interact with both the delivery and receipt of the intervention. The challenge is not just to include these additional elements in a trial of an intervention but to understand the intervention as a whole in terms of how it produces outcomes and how the different elements are causally related to each other. This involves understanding a complex set of processes and events and how they are linked together in causal pathways. These causal pathways articulate the underpinning theory(ies) of the intervention, as the theory of how an intervention works necessarily explains how the intervention causes change. For example, an intervention that uses a food diary to promote weight loss might be based on a theory of self-monitoring of dietary intake and how this affects eating behaviour, as well as a theory of how diaries might be best designed to encourage participants to use them. A description of the causal pathways would explain how the food diary design and delivery influenced participants to engage with the intervention, how the food diaries were actually used and influenced dietary behaviour in participants, whether and how self-monitoring occurred, contextual factors such as family mealtimes, and how these factors contributed to any weight loss in participants (or not). Process evaluations are challenging because the interventions they analyse usually have several layers of complexity due to the challenging problems they are trying to address, the many elements and processes that may occur during the intervention and which are likely to interact with each other, the different levels in which they operate and interact (e.g. intrapersonal, family, and community), and the number of outcomes they may be trying to bring about [25]. One weakness of quantitative research can be the tendency to fill in gaps in explanation with the researchers’ own assumptions. This occurs because of the distance between the researcher and the data in quantitative research [26] or because statistical results only show association between two variables, and researchers may attempt to fill in the missing explanation of how the intervention worked with speculation [27]. Traditional epidemiological methods can establish relationships among variables, but these are not able to fully explain why outcomes occur. As a result, the understanding of processes in an intervention may be flawed or incomplete. Qualitative methods, such as interviews and focus groups, make an important contribution to process evaluations because they can produce rich, detailed information about processes, contexts, and causal pathways in ways that quantitative data cannot. For example, interviews with participants can capture rich narratives describing how individuals experience and react to an intervention and why they may change their behaviour as a result (or not). Qualitative methods also capture the depth and complexity of topics, and are flexible enough to capture unexpected data which may further contribute to the understanding of how interventions operate. All of these aspects of qualitative data are necessary to develop the comprehensive understanding of how interventions operate, which a good process evaluation will aim to produce; furthermore, these aspects contribute to theory-development in the field through explaining causal pathways occurring in interventions. This article argues that ethnography has further strengths over and above the benefits of standard qualitative methods, just outlined here, that could improve process evaluation methods. First, ethnography can be useful in acquiring valid data on intervention processes through collecting data in naturalistic settings and through observing behaviours and events as they occur in real-life settings, rather than through post-hoc interviews and self-report. Second, ethnography collects and analyses data in a way that is inherently embedded within the subjects’ cultures and social worlds and could thus contribute to incorporating contextual factors of the delivery and receipt of interventions. Third, ethnography is an iterative, theory-building approach which is ideally suited to working with and developing theory, another important function of process evaluations that contribute to wider knowledge building within a field. The way in which ethnography is best employed in process evaluations is partly dependent on the type of trial being conducted. In process evaluations for feasibility trials, ethnographic methods could be employed to assess trial methods or to develop the intervention - especially for unfamiliar contexts or hard-to-reach groups - in order to inform a definitive trial. In the ethnography of Garcia et al. [28], which was an intervention for prophylactic HIV medication for black men who have sex with men (MSM) in the USA, the study was used to investigate what ‘usual care’ was in order to successfully integrate the intervention with ‘usual care’, and also uncovered factors such as mistrust of medical services and medications among participants, which helped inform how the main trial was designed [28]. In definitive trials, ethnography could be used to develop and build intervention theory, explain why different outcomes occur for different subgroups, or explain recruitment or retention issues. The three ways in which ethnography can be applied to improve process evaluation methodology, and their applicability to feasibility and definitive trials, are discussed below.

## Validity of data

The quality of a process evaluation, as in any research, is dependent on the quality and validity of the data collected. Data validity is defined here as the closeness of the relationship between the data collected and reported and the phenomenon being studied. In the case of process evaluation, valid data may be required on the processes of intervention delivery, intervention receipt, mechanisms of impact, intervention contexts, and causal pathways. Threats to data validity in process evaluations arise when data are collected from stakeholders and participants when time has elapsed or when factors such as social desirability bias influence how the data are reported to researchers. Process evaluations often collect data from individuals who are delivering the intervention as well as from the intervention recipients to analyse how interventions are delivered and received in different settings. This may include evaluating intervention fidelity (whether the intervention has been delivered as it was designed to be delivered). Self-report biases may occur from those who deliver interventions because they are reporting their own work and performance and, therefore, may present a positive version of events. Self-report of health behaviours by participants can also be subject to bias; for example, participants may report consuming fewer calories or units of alcohol than they actually did. Data may also be collected about retrospective experiences, often through interviews, over the course of an intervention for example, and participants may not remember their experiences or behaviours accurately. Furthermore, only selected data may be reported to the interviewer by trial participants, depending on how a participant views circumstances; this filtering is inevitable and can be interesting in itself but necessarily limits the data that the researcher has access to and its validity.

Ethnography studies social and behavioural phenomena in naturalistic settings through participant observation, where the researcher is embedded in a social world and, thus, uniquely observes behaviours as they occur in situ. Observation as a key method of ethnography has several benefits. First, data collection is direct rather than being reported at a later time point in interviews or focus groups and is unmediated through participant interpretation or the passage of time. This partially overcomes the problem where practitioners and participants may not remember or report their behaviours in an unbiased way for various reasons, such as practitioners presenting a professional image to researchers or participants constructing their own narratives retrospectively (they may, of course, adjust their behaviours in response to ethnographic observation; this will be discussed below). Second, social groups are observed directly in ordinary, everyday settings of participants; this method is useful for understanding how people delivering or receiving an intervention behave in real life, both in settings where interventions are received and in family or social settings where health behaviours occur or where new behavioural skills are enacted. This can be valuable for hard-to-reach groups, such as substance abusers, or situations such as youth drinking in town centres [29], as some health behaviours only occur in specific settings. Third, the connections between different data on behaviours, events, contexts, and so on can be observed, rather than being collected atomistically as separate, unrelated items. Knowing these relationships can be helpful, for example Pavis and Cunningham-Burley [29] sought to understand the risk behaviours of young men hanging around on the streets of a Scottish town. They argue that previous research on this topic tended to focus on the individual risk factors associated with the uptake of behaviours such as smoking, drinking, and illicit drug use, whereas they sought to understand these behaviours within the context of young people’s broader lives and culture. They describe, for example, how cigarette smoking behaviour in young people was related to different types of interaction within social groups, which served to initiate and reinforce social bonds. This study was not a trial, but this type of information could be used for a process evaluation; for the example just outlined above, the researcher could incorporate questions into the interview about social bonds and how these are affected by quit attempts or further observation on how quit attempts interact with the management of social bonds in peer groups. This type of information would enhance the ability of a process evaluation to explain how a smoking intervention operates in conjunction with the social practices of smoking and any effect on trial outcomes.

Ethnography, because it uses observation as a central method, has an advantage in overcoming problems such as self-report that exist in other qualitative studies which only employ interview and/or focus group methods. Nonetheless, disadvantages such as bias exist in all methodologies, including ethnography, and researchers commonly take measures to minimise them. However, an additional benefit of ethnography is that it usually employs multiple methods, and this approach tends to balance out the strengths and weaknesses of each method. Ethnography does this not just by using more than one method but by integrating them in the analysis; this is not always the case in other types of ‘mixed methods’ studies, including trials that incorporate qualitative studies [30].

The ethnographer collects naturalistic data through ‘participant observation’, which means that the researcher must acquire the status of an insider and become part of a social group to some degree to observe and experience life as an insider would. This makes the method distinct from just ‘observation’. In order to collect data through participant observation, the researcher must first gain entry into a social world and also gain acceptance there. Entry is achieved through good access, which is ultimately dependent on establishing trust and rapport with one’s research subjects. This will ultimately affect what the researcher is told by members of the social group and what the researcher is allowed to observe. For ethnographic research, the establishment of trust can rely heavily on local sponsorship from an influential member of the social group; it can be very difficult for a researcher to approach a group ‘cold’. Often these key members become ‘key informants’, vouchsafing for the ethnographer’s credibility, facilitating introductions, and ultimately steering the ethnographer to interesting insights. Access negotiations, however, are not merely confined to the early stages of the ethnographic project, but rather ethnographers would be wise to continually monitor their on-going relationships with the collective. Key informants may also be ‘gatekeepers’ to the research setting, although other, more formal, gatekeepers (such as head teachers or department managers) may be present, whose approval and support is required in order to collect the data, but they may not be members of the social group being observed. Access can also be facilitated by the fact that naturalistic observation may introduce less disruption to the setting compared to interviews or focus groups (no arrangements have to be made for people to attend interviews or focus groups, and research participants do not have to take time out of their other activities to attend), and therefore be more acceptable to gatekeepers and participants, who may include busy health professionals trying to deliver services [31].

Once participating in a social world, the ethnographer uses participant observation as a tool to collect data which is occurring naturalistically; this may include reflecting on how they experience phenomena themselves as an ‘insider’, as well as observing others’ behaviour. Interviews will also typically be conducted, and these can be compared to observational data. Ethnographic interviews are likely to have improved validity because they are conducted by an ‘insider’ who understands the social world, and participants also have an awareness of the ethnographer’s familiarity with their social world. Benefits exist to the researcher becoming accepted in terms of the amount and quality of the data the researcher is likely to collect as the researcher gains trust and acceptance and becomes a normalised part of the environment. The quality of interviews can be improved by a better understanding of the researcher and the researcher’s ability to focus questions on the most relevant factors, while gaining trust from the participants to engage in and answer interview questions fully. Wight [32] and his co-worker spent 3 years living within a working-class, industrial village, and although their participation in the community was initially self-conscious and awkward, they eventually became accepted by most of the community. Acceptance may be challenging if the researcher is perceived as very different from the community they are studying: social distance between the researcher and a group or community can result in lack of trust or not knowing enough about the phenomena under study to ask the right questions. An additional factor in process evaluation is that the researcher may be closely associated with the trial team and the intervention (and therefore as someone who has an investment in whether the intervention is successful) and may have to distance themselves from the main trial for a period in order to become an ‘insider’ in a group or community. Overcoming social distance to acquire the status of an insider may, therefore, take varying degrees of time and commitment to a social group. Because they have become part of the group and are less visible as an outsider (although this process is never complete), the role of the ethnographer as an insider thus reduces the Hawthorne effects of observation. This is similar to passing the ‘dead social scientist test’ [33], where the researcher reflects on whether the data collected would have been the same if they were dead (i.e. not there). There are challenges in ascertaining the validity of data, even within ethnography, because the researcher will always interrupt the natural setting. However, this effect is usually reduced over time due to the longevity of the presence of the researcher in the field because it is difficult for participants to maintain pretence for a long time [34]. If the ethnography is only conducted in the intervention arm, it could potentially bias the trial findings because it is quite an intensive method; balancing the tension between data collection and the need for trial findings to be unbiased and have external validity is a common issue for process evaluation and needs to be considered carefully in the research design.

It is clear through these descriptions of participant observation that ethnographers use themselves as a research instrument to a greater degree than in other methodologies; this will have an impact on the research data. Ethnography recognizes the work of interpretation of the ethnographer in producing an account of a culture, both in influencing the research setting (although, as discussed above, this is minimised as much as possible) and in the analysing and writing up of the data. The ethnographer, who usually works in isolation, brings with them their own values, beliefs, and experiences to all stages of the research. It would be impossible, indeed undesirable, for the researcher to be uncontaminated by their own background; instead, the researcher may reflect upon and acknowledge how one’s beliefs and background could influence the study. The activity of being mindful of one’s effect on a setting in how people behave and one’s own personal contribution to the interpretation of data is termed ‘reflexivity’ [35]. Ethnographers and qualitative researchers use reflexivity to account for their roles in the production of the research data, reporting on this in their findings, and thus make clear to the reader how they have influenced the data. Reflexivity is a practice adopted by ethnographers to manage their position as both an insider and an outsider in order to account for their role in the production of data. As reflexivity is an essential part of the ethnographic process, ethnographic writing tends to provide accounts of the researcher in the research setting and their reactions to it, combining subjective narrative with ‘thick description’ [36] (discussed below). Since field notes and ethnographies are authored products, they cannot be treated as straight-forward objective representations of the setting: rather, they are selective in what they choose to describe and how it is portrayed. Critical anthropology also emphasises that the output of an ethnography is a combination of the researcher’s perspectives and categories and that of the community they study. While reflexivity as a term or a practice may be somewhat unfamiliar to positivist researchers, this is a good practice in any field of research. Our perspectives and questions shape the data we choose to collect and, therefore, what our findings are, whatever our method or research paradigm. Further, most trialists are aware that trials affect how interventions operate and that their findings will show what an ‘intervention + trial’ produces rather than just the intervention. Being reflexive about this effect of research activity means findings can be interpreted in the light of transparency about the role of the researcher.

## Social context

Health-promotion research has increasingly recognised the importance of the social determinants of health, such as socio-economic status [37]. Attention has also turned to community and legislative environments, such as the availability of local parks or smoking bans, and their influence on attitudes and behaviours. At the intervention level, increasing recognition exists that interventions have an impact on and are affected by the contexts in which they operate; for example, Hawe et al. [38] argue that interventions are events in a system rather than a closed process. Because the unit of analysis of an ethnography tends to be a particular social world, this methodology addresses social contexts in a holistic way, in which data are related to contextual features and events. Hinder and Greenhalgh [39] and Schoenberg et al. [40] explore the role of the family context, employment, and stress and how they influence the management of diabetes in this way. In the Hinder and Greenhalgh study, family members were found to be involved in blood sugar monitoring and dietary management to varying degrees, from providing sugary food in the home environment to putting pressure on the individual with diabetes to maintain good control over their condition [39]. Schoenberg et al. consider patients’ accounts of stress from difficult family situations or jobs and how the participants attributed behaviours such as poor eating habits to this stress [40]. This type of understanding of what triggers different types of health management can be critical information for a process evaluation because it enables the researcher to understand the various contextual factors which affect participant behaviour in response to the intervention and ultimately their outcomes.

Ethnography adopts a broad approach to ‘context’ in that it also includes the cultural environment. This is an inherent and distinctive aspect of the ethnographic approach [41]: the benefits of ethnographic methods over and above qualitative studies, however extensive they may be, are sensitivity to the socio-cultural environments and in their interaction with human sense-making, beliefs, values, and behaviours. This perspective is often addressed through the use of the participant observation method for situations where it would be difficult to uncover cultural issues and social norms to the same extent in individual interview studies. Behaviours are not described in isolation in an ethnography but are accounted for in relation to other aspects of a culture, requiring a particular analytical approach to social life, including an account of the subtle patterns and rules of social behaviour. This may include the details of everyday life and habits, verbal and body language, taboos, humour, dress codes, rules of behaviour, food culture, material and visual culture, rituals, and rites of passage (formal and informal). Behague et al. [17] describe how ethnographic methods revealed local terms for medical conditions in Brazil, which subsequently improved survey data validity. Additionally, they sought to understand the influence of family pressures, school experiences, and perceived social status on teen pregnancy. In another example, Nelson et al. [42] found that in an intervention designed to promote open communication between adolescents and parents about sexual health in Latin America, some men viewed open communication about sexual behaviour as being ‘for gays and women’. This type of contextual information may not be immediately apparent in a research site but can be essential for producing a valid and comprehensive account of the acceptability and receipt of an intervention.

Ethnographic research emphasises the need to learn the sub-culture of the people being studied and to interpret the world in the same way as they do. This perspective is best understood by a researcher through a prolonged immersion in the setting or to gain, as Geertz recommends, ‘close in contact with far out lives’ [43]. Learning a culture does not just mean detailed knowledge of the activities or practices the people engage in but also the significance of the activities or practices. Pavis and Cunningham-Burley describe ‘culture’ as referring to the ‘shared stocks of knowledge, values, ideas and systems of meaning that are held collectively*’* [29]. This can be a valuable way to understand why participants respond to interventions in the way they do, as their ‘rational behaviour’ from within their context may be quite different from the researcher’s behaviour [15]. For example, Wight’s [32] exploration of material lifestyles in an industrial village in central Scotland emphasised the interconnectedness among drinking, masculinity, and employment and the strategies that men developed to retain their self-esteem in a period of recession. Rationality can be locally or culturally specific; this can be true both of professionals and patients [15]. Schoenberg et al. [40] highlight the significant differences in the way professionals and patients understood diabetes management in the USA, with patients describing stress within their social context as a significant factor and its impact on self-care, whereas professionals engaged with this factor only minimally. Close contact and deep understanding of the social worlds within which trials are conducted can help focus process evaluations to ask the relevant questions and observe the key processes that are likely to affect the success or failure of the intervention.

Ethnography is also a useful methodology for examining the specificity of particular social contexts. Traditionally, a European anthropologist would have studied a culture in a remote and ‘exotic’ area such as a Pacific island, whereas now, ethnographies might be of groups of teenagers who drink on the streets or users of internet forums. Organisations, such as schools or GP practices, are also social worlds of interest because many interventions are delivered through them (see Table 1). Organisations have their own cultures, and professions have distinct practices which affect how an intervention is delivered.

**Table 1:** Using ethnography to investigate implementation in a healthcare setting

Jansen et al. [58], reporting on an ethnographic process evaluation of a pragmatic trial of multidisciplinary patient care for patients in Rotterdam and The Hague, described ethnographic insights into intervention implementation problems in primary healthcare centres. The process evaluation uncovered barriers such as (1) practice nurses requiring more time to organise the implementation of the intervention and to coordinate with colleagues in the health centre; (2) peer educators, who were delivering health education to some patients, were not allowed to access records in one GP surgery due to a decision by GPs about who could access patient records; (3) GPs were unwilling to change their schedules to accommodate the intervention; and (4) the assistants, whose roles were to support the intervention, were moved to other tasks due to resource constraints. As a result, the researchers produced intervention guidelines to overcome these barriers.	

Huby et al. [44] describe two ethnographic studies which, in order to identify how care could be improved, contrasted patient and provider perspectives on the complexity of networks of care provision, highlighting problems such as poor liaison and gaps in care and how this impacted patient well-being for HIV and stroke patients. This type of analysis could be easily applied to process evaluations which examine implementation processes during trials, partly to inform how the intervention might be rolled out more widely after the trial.

The emphasis of ethnography on social context may be particularly useful during feasibility and pilot trials in which interventions and data collection methods are being developed, especially in cases where social contexts are not well understood; ethnographic data could help researchers understand whether a trial or intervention is likely to work well and be acceptable to participants in a particular type of organisation or setting. Maher et al. [45] reported on a feasibility study for a Hepatitis C vaccine trial for intravenous drug users, where ethnographic data uncovered the importance of altruism and attitudes towards financial reimbursement in the willingness of users to participate in the trial and also produced useful information for improving communication with participants. This approach could also easily be used for intervention development. For example, Hong et al. [46] used an ethnographic process evaluation to develop culturally and socially appropriate communication tools and a message diffusion programme for an HIV prevention programme for an ethnic minority group.

## Theory building

Process evaluations should be based on the underlying theory of an intervention in order to structure the data collection and analysis around how the intervention is hypothesised to operate [24]. An RCT is a theory-testing endeavour, where the primary (statistically expressed) outcome indicates whether the hypothesis that the intervention will change behaviour and improve health is proved or disproved. Process evaluations also have a role in further developing theory since they can be used to (deductively) test an underpinning theory about how an intervention works but may also (inductively) refine theory or even build new theory by collecting exploratory, qualitative data. Ethnography is a methodology which works closely with theory and, like other qualitative methods, has the ability to adapt to emerging research questions. Because of this flexibility, it can be used to address new or emerging research questions during a process evaluation and, in this way, can contribute to theory development [47]. While new theory may emerge or develop during the feasibility stages of studies, and because definitive trials are longer and usually employ a more complex set of mixed methods, more opportunity may exist to develop theory at this stage. Ethnography is characterised by three key features which facilitate theory testing and, in particular, theory building: an alternation between emic and etic perspectives, a flexible and iterative approach to data collection which follows emerging themes, and ‘thick description’.

Ethnographers develop theory by operating with both an insider view as well as an outsider view, defined as ‘emic’ and ‘etic’ perspectives. The emic perspective is the explanation of the social world provided by those within the cultural group; data from this perspective are collected through the ethnographer’s ‘participant observation’ method and by gaining the status and perspective of an insider, described above. The etic perspective is that of the analyst, or the researcher. The ethnographer deals with both perspectives by alternating between the roles of ‘native’ insider and ‘naïve’ outsider. An ethnographic account includes not only the emic description of the beliefs and perspectives of members of a social world but also an etic, a theoretical description which attempts broader and more abstract conclusions about the social world. It is this etic perspective from which an ethnographer builds theory, moving from description to conceptual analysis to theory. The social distance of the ethnographer as an outsider can thus be used to the ethnographer’s advantage, allowing the ethnographer to maximise the anthropological position of strangeness and not to take for granted what a member of the community would consider unremarkable.

Ethnography tends to use different methods iteratively as a study progresses, rather than relying on formal data collection schedules. Ethnographers adopt a flexible approach to research design, responding to ideas as they emerge during a study, and use iterative data collection as an opportunity to validate data and test ideas and hypotheses, for example by triangulating data [41]. Data are typically recorded through observational field notes, which allow the collection of relatively concrete descriptions of the setting and the activity or whatever is considered suitable and useful. Ethnographers typically then employ other methods, often interviews, and develop further methods over time. For example, Bunce et al. [31], in an ethnographic process evaluation of a technology-based diabetes intervention in health clinics, used diaries, document analysis, interviews, group interviews, and a survey in their data collection. Emerging topics and themes can be followed up using appropriate methods, in order to build concepts, ideas, and theories. Furthermore, because of the exploratory nature of this methodology, an ethnographic study can uncover and follow up novel data in order to build theory. Unexpected data might include data available from a source in the field that had not been anticipated (for example, a voluntary organisation keeping records of client feedback that the researcher was not originally aware of). Alternatively, data might be unexpected in that the data collected might look very different than anticipated: if a study was exploring an intervention to promote walking to work and the main study was concentrating on aspects such as road layouts or pedestrian crossings, the ethnographic data might reveal that there was a history of crime in the locality or a culture where travelling by car denoted high social status that were the primary reasons for not walking to work. Although unexpected data can create resource or time-tabling problems for a study if it takes an unpredictable turn, finding unexpected data can be one of the most useful and interesting parts of a study, as it produces new knowledge which may confirm, build, expand, or improve on existing theories in some way. It challenges researchers’ assumptions and means findings could be a useful departure from ‘received wisdom’ in the field towards more valid theory.

Once data have been collected in this way, ethnography then builds theory through ‘thick description’, one of the key terms associated with ethnography [36]. According to Geertz, ethnographers must present a report which is composed not only of facts but also offers commentary, interpretation, and meta-interpretations. By contrast a ‘thin description’ would be a superficial, descriptive, and factual account lacking interpretation and failing to explore the underlying meanings, intentions, or circumstances of actions. Thus, thick description is not simply a matter of amassing and presenting relevant detail, but it is the interpretative characteristic, rather than the detail, that makes the description thick [48] (see Table 2). For a process evaluation, a thick description could be employed to account for the processes occurring in an intervention – such as the patient-provider relationship, acceptability, participant agency and response to the intervention – which help explain how outcomes are achieved [40, 49, 50].

**Table 2:** ‘Thick description’ of an intervention and its social context

Nelson et al.’s [42] ethnographic study of a intervention to promote communication between parents and their adolescent children about sex and sexual health in Latin America describes how an intervention was premised on the notion of ‘open communication’ being a ‘good thing’ but was often interpreted by participants as *confianza* (trust), which may or may not include open forms of communication. It also describes the various ways in which language was used between adolescents and parents, for example by parents to exert power over their children’s sexual activity, or by adolescents to resist this; and how community members expressed social norms about what was acceptable sexual behaviour for men and women, which could lead to contradictory statements being made to young people (because mothers and fathers would make different types of statements, for example). The interpretation of the intervention and the different functional uses of language (besides just ‘communication’) were analysed in terms of their embeddedness in social and cultural norms, as was the international development intervention itself and its intention to change communication behaviours.	

## Limitations

The ways in which ethnography can contribute to process evaluations have been outlined here, but as with any method, ethnography also has limitations. The first characteristic of ethnography that might strike a researcher (especially one writing a funding application) is that ethnography can be time-consuming and, therefore, expensive. The researcher has to gain access and then observe a social world with a certain degree of intensity and over a period of time. This has obvious implications for resources for a trial; often, not every site can be visited by the researcher, and therefore, decisions have to be made about how to sample sites. However, fieldwork for a process evaluation does not have to be as long as traditional ethnographies; applied ethnography is typically shorter [14], and ethnographic methods can be adapted for a study. Shorter ethnographies could be conducted in order to produce timely findings, for example, to influence the design of a definitive trial [28] or to inform policy makers about important contextual factors for implementation at the end of a trial. Even so, ethnography will still be a resource-intensive method because of the researcher time required. However, thorough process evaluations which collect valid data are important for understanding how interventions work and for avoiding expensive failures of theory and interventions in the future; resource justifications can be made on this basis. Furthermore, while a longer-term iterative ethnography might be a more expensive study, this may be cheaper overall than a study that produces limited findings and then requires a second, follow-up study to explore issues further.

Second, because ethnography is usually an in-depth study of one social world, limitations exist to its generalizability. However, ethnographic research sites are often chosen for their ability to generate interesting theoretical insights or information about a sub-group, rather than necessarily being chosen for their typicality. Alternatively, the theory generated through an in-depth study may have wider applicability, and this kind of research, therefore, often has ‘theoretical generalisability’ [51]. An in-depth study of a phenomenon, through its close study and uncovering of processes that other methods might miss, produces theory that can then be investigated in other cases to explore how universal that phenomenon is. In any case, a process evaluation within an RCT will also have limited generalizability even if it is conducted across several sites because it will necessarily take place in some contexts or for some populations rather than others. RCTs themselves have limited generalisability because they are conducted within a limited population and at a particular time point. Therefore adopting ethnographic methods for a process evaluation will not make a trial less generalizable than it already was, unless it focuses on a sub-sample or case within the trial population.

Third, depending on the setting, ethnographic methods may produce additional risks for the researcher because field dangers may include physical violence, emotional strain, and danger arising from ‘guilty knowledge’ of illegal activities [52]. This could occur during observations of binge drinking behaviour in city centres, for example. Ethnography faces the same risks and challenges as qualitative research; in ethnography, however, researchers are often slightly more likely to place themselves in risky situations because it can be more difficult to anticipate the nature of the fieldwork in comparison to an interview study, partly because fieldwork can take place in less structured or formal environments. Bloor et al. [53] highlight risks for researchers in the field, particularly for ethnographers exploring dangerous settings. They point out that risks are often left to individuals to manage, rather than institutional structures such as ethics committees. They discuss institutional ways to mitigate risk, including risk assessment, researcher management, resources for safety, and insurance. These can be included in protocols and SOPs for process evaluations, as well as in institutional policies.

A fourth issue is not so much a limitation as an additional consideration: that is, the ethical issues which arise from the ‘insider/outsider’ role of the ethnographer. One could argue that ethnographers have a tendency to deviate from the formal rules of ethics that have been widely accepted by other social science researchers [54]. For example, in order to maintain fieldwork relationships, ethnographers are likely to present themselves during access negotiations and data collection as more sympathetic to the behaviours, beliefs, and social values of the community than they might actually be. A tendency may exist for ethnographers to partly conceal their real motives for conducting the research in order to ensure that members of the community do not adapt their behaviours and beliefs. This can be a particular issue if the research focuses on clandestine activities such as drug use. In practice, however, ethnographies typically portray sensitive accounts of social worlds with sympathy because they communicate a groups’ insider perspective. In addition, community members may not fully understand or remember the details of the ethnographic research and maybe be unaware at times that the researcher is collecting data; in our experience participants tend to forget the details of methods that have been explained to them and also tend to associate ‘data collection’ or ‘research’ with questionnaires or interviews rather than observation. This has implications for informed consent, and the regular negotiation of ethics, rather than a contractual agreement at the outset of the research, may be more appropriate in order to manage this [55].

Finally, a more pragmatic limitation of ethnography is that the flexible, iterative data collection and analysis process of this methodology is at odds with what funders and ethics boards usually require in the planning stages of a study [56]. Whilst a flexible, iterative, and exploratory approach is a strength of ethnography, it ultimately makes it more challenging for ethnographic research to be funded and approved. Funding bodies and ethics committees require detailed research plans and schedules, including what the fieldwork will involve, how many interviews will be conducted, where observation will occur, what type of data will be collected, and what the focus of data collection will be. Some decisions about data collection may occur later on in an ethnography, rather than being planned in advance. Similarly, ethics may be agreed on with the community on an ongoing basis, as noted above; this does not fit well with the contractual model of consent usually used in studies such as RCTs and approved by ethics committees in advance of the study. Taken together, these limitations of ethnography and the responses they require highlight how distinctive ethnography is as a method and how resources, research planning, safety, and ethics all have to be considered within a different framework from standard RCTs.

## Conclusions

Process evaluation is a developing area of complex interventions research. Continued recommendations to conduct process evaluations alongside RCTs [2, 24, 25] have meant that new methodologies are required to address the challenges involved in explaining how interventions work and trial outcomes are achieved. Ethnography is ideally suited to the challenge of process evaluation because it produces data with high validity, inherently incorporates social contexts, and works closely with theory to develop an understanding of how interventions work. Trials being conducted in novel or complex social contexts for hard-to-reach groups or in cases where data validity or theory-building are key issues should consider adopting ethnographic methods in the process evaluation. The strength of incorporating social contexts may be particularly useful for process evaluations in feasibility trials, whereas ethnography’s strength in theory building may be especially relevant for definitive trials. However, ethnography is a relatively unfamiliar method in the world of RCTs and requires a distinctive approach to research design and quality criteria (such as the appropriate use of reflexivity), which may be unfamiliar to trialists more used to quantitative research perspectives. It also requires, as with process evaluation more generally, adaptation to the research question, type of trial, and level of resources available. A consideration of when findings are produced and how they inform future studies depends on factors such as whether the process evaluation is for a feasibility or definitive trial; this is a broader issue affecting the use of qualitative methods alongside trials [57].

This article has taken a largely pragmatic perspective in demonstrating ways in which ethnographic methods could be helpful in process-evaluation methodology. Where ethnography is used in a very constructivist way, the analysis may be difficult to combine with an RCT’s findings if the trial’s overall methodological approach is towards positivist approaches to data. However, critical realist perspectives take into account the fact that data are always mediated by interpretations of the participants (and researchers), while still attempting to produce an account of how an intervention works that could be considered an approximate account of the ‘real’. Most researchers working in trials and process evaluation fields, in our experience, are relatively pragmatic in their approaches to the qualitative/quantitative debate and also accept realist approaches, as expounded in the work of Ray Pawson for example [27]. Reflexivity, as discussed above, can be useful in managing the tension between acknowledging the interpretation that goes on in producing data and in trying to present a valid account of how an intervention works in a given context.

As well as the benefits that ethnography brings in terms of a methodology, the value of ethnography as a product should also be considered. This is normally an account of a social world and an ethnographer’s time spent there. It usually has a narrative element, which can be an accessible and engaging way to draw people into reading about the research. Its ‘thick description’ of a social world can also be very interesting, as it highlights distinct features of interest, illuminates different personalities, and shows how a social world fits together. This has implications for the impact of research, since the human interest in a story or a social world can help the research team communicate a rich account of their findings to an audience.

## Abbreviations

MRC: Medical Research Council
RCT: randomised controlled trial
